# A Mixed Methods Exploration of Surf Therapy Piloted for Youth Well-Being in Post-Conflict Sierra Leone

**DOI:** 10.3390/ijerph18126267

**Published:** 2021-06-10

**Authors:** Jamie Marshall, Sallu Kamuskay, Michaella Margaedah Samai, Isha Marah, Fanta Tonkara, Josephine Conteh, Sullayman Keita, Oullematu Jalloh, Mohamed Missalie, Mohamed Bangura, Olufemi Messeh-Leone, Messeh Leone, Brendon Ferrier, Russell Martindale

**Affiliations:** 1 School of Applied Sciences, Edinburgh Napier University, 9 Sighthill Court, Edinburgh EH11 4BN, UK; b.ferrier@napier.ac.uk (B.F.); r.martindale@napier.ac.uk (R.M.); 2 United Sierra Leone, 9 York Road, Sussex Beach, Freetown, Sierra Leone; sallukamuskay@gmail.com (S.K.); Mworldsafe001@gmail.com (M.M.S.); 3 Pipul Pikin Foundation, 60 Maxwell Street, Wellington, Freetown, Sierra Leone; pipulpikin@gmail.com (I.M.); salonemessenger@gmail.com (O.M.-L.); 4 Moseray Fadika Trust Foundation, 154 Wilkinson Road, Freetown, Sierra Leone; teamfadika@gmail.com (F.T.); missalie7@gmail.com (M.M.); 5 Job Opportunities for Youth (JOY), JOY Centre, 38 White Street, Tokeh Beach, Freetown, Sierra Leone; joycentre.sl@gmail.com (J.C.); mohamedsbangura@gmail.com (M.B.); 6 Young Leaders Sierra Leone, AYV House, 3 Wesley Street, Tower Hill, Freetown, Sierra Leone; theyoungleaders.sl@gmail.com (S.K.); oullematuprincessjalloh@gmail.com (O.J.); 7 The Messeh Leone Trust, Malaika Chambers, 17 Off Kingharman Road, Freetown, Sierra Leone; messehproject@gmail.com

**Keywords:** surf therapy, mental health, sport for development, mixed methods, evaluation

## Abstract

Young people in post-conflict and post-epidemic contexts such as Sierra Leone face a range of mental health challenges as part of their daily life. An innovative approach to Sport for Development that could offer support to youth mental health is surf therapy. This research used an uncontrolled mixed methods approach to explore surf therapy pilots run by five youth-focused and community development organizations around Freetown. Four sites provided useable pre/post data using the Stirling Children’s Well-Being Scale (*n* = 58, average age = 12.9). Three sites were associated with significant (*p* < 0.017) large effects (*r* = 0.65–0.84) on participant well-being. One site was associated with a non-significant (*p* < 0.380) small negative effect (*r* = −0.22). A synthesis of qualitative data within the five evaluations triangulated with quantitative findings and provided important context in terms of challenges to service delivery. This included low attendance as a plausible mediator for why one site saw very different results than other sites. Combined, these processes highlight the need for future research exploring possible dose-response relationships in surf therapy. This study also provides a foundation for more rigorous research in the future. These promising findings support continued and optimized delivery of surf therapy in Sierra Leone to support youth mental health.

## 1. Introduction

Armed conflict such as civil war has been associated, both directly and indirectly, with negative mental health among children and young people [1]. The intergenerational nature of conflict-induced negative mental health means it lasts long after conflicts have officially ended and in fact spreads within family dynamics [2]. This means young people who took no active part in historical conflicts are still experiencing associated negative mental health. Between 1991 and 2002, the West African country of Sierra Leone experienced devastating civil war, defined by widespread human rights abuses and collapsing infrastructure. During this conflict, nearly 7000 children are believed to have been recruited as child soldiers [3]. The country also experienced an Ebola epidemic between 2014 and 2016 that killed nearly 4000 people [4], with a compounding negative impact on mental health [5]. Priority areas for improving youth mental health in Sierra Leone have previously been identified as improving positive peer support, delivering effective coping/self-regulation skills and knowledge, and providing alternatives to risky behaviors [6]. The Messeh Leone Trust (https://www.messehleone.org/ (accessed on 5 June 2021)) recently brought together a collection of youth and community development organizations in Sierra Leone to pilot surf therapy within Sierra Leone and specifically address these priority areas.

Surf therapy in Sierra Leone is an example of Sport for Development (SfD), with an explicit focus on mental health and well-being. There remains a lack of rigorous evidence pertaining to the efficacy of SfD [7,8], including SfD’s effectiveness in supporting positive mental health in post-conflict contexts [9]. Despite the lack of rigorous evidence around SfD in supporting post-conflict youth mental health, promising results can be seen within organizational evaluations and other non-peer reviewed materials. A recent review of the evidence on the effectiveness of surf therapy, including supporting youth mental health, was encouraging [10] but was hampered by a lack of rigor, especially in terms of control groups and sample sizes. No research has been carried out into the effectiveness or associated impact of surf therapy for youth mental health within post-conflict settings. Further research is required to determine the effectiveness and associated impact claims, both for surf therapy and within the wider SfD paradigm, especially around youth mental health.

The only research that has currently been carried out on surf therapy within the post-conflict context explored initial program theory in the Waves for Change (W4C) intervention in neighboring Liberia [11]. This study highlighted the creation of a safe space, positive social support, effective transfer of coping skills, and respite from negative emotions while in the water, as integral to the intervention achieving its associated outcomes. These elements of the intervention align with previously mentioned priority areas for youth mental health in Sierra Leone [6]. The findings also triangulate with studies exploring surf therapy for youth mental health in different contexts [12], especially around the importance of a safe space, social support, and respite. The initial program theory expounded within the research carried out in Liberia provided the theoretical framework for the piloting of surf therapy in Sierra Leone. The aim of this study was to provide a mixed methods evaluation of the associated impact of a range of surf therapy programs across Sierra Leone.

## 2. Methods

### 2.1. Surf Therapy in Sierra Leone; The Wave Alliance

This study is based on evaluation reports produced by 5 separate youth-focused and community development organizations who collaborated to deliver surf therapy pilot projects near to Freetown, Sierra Leone. The 5 organizations involved were the Moseray Fadika Trust (Site 1), Young Leaders Sierra Leone (Site 2), United Sierra Leone (Site 3), the Pipul Pikin Foundation (Site 4), and Job Opportunities for Youths (Site 5). The organizations made these evaluations publicly available online, and links can be found in the Appendix A. All of the organizations already provide youth support around education, employability, and other contextual challenges. The organizations were brought together by the Messeh Leone Trust (MLT) in 2019 to collaborate and bring surf therapy to Sierra Leone for the first time. MLT also provided technical, financial, and programmatic support to partners on the ground in Sierra Leone. This support included helping with setup, securing safe spaces/sites, delivery of material support, and monitoring of pilot projects. MLT also helps coordinate evaluation support from third parties to enable the objective, impartial evaluation of projects. The initial step was an approach by the organizations to the Wave Alliance (https://www.waves-for-change.org/the-wave-alliance/ (accessed on 5 June 2021)), an incubator program that offers training and ongoing support to start-up surf therapy organizations within Low-to-Middle-Income Countries (LMICs). The training is centered on mental health theory, program design and implementation, staff hiring and training, fundraising, and evaluation. The Wave Alliance framework is based on the Waves for Change model of surf therapy and includes recent research carried out into the intervention’s program theory [11]. While based on this model, the training is not prescriptive and is designed to provide trainees the flexibility to best address the challenges of their own context, encouraging the generation of new program content. Upon completion of a 2-week training program in Cape Town, the organizations from Sierra Leone returned and implemented small surf therapy pilots, running from November 2019 to March 2020. Sessions commenced in January 2020 and ran for 10–12 weeks (depending on the site), with participants attending surf therapy once a week for two hours. Sessions consisted of an initial meeting, with a safe space held so all participants could ‘check in’ with how they were feeling. This was followed by a warm-up game and the learning activity for the day. Activities were based around surfing, but were designed to support emotional regulation, goal setting, mindfulness, positive socializing, and respite [11]. The session finished with time allocated to unstructured but supervised surfing/swimming, followed by a debrief. It is important to note that these learning activities, the safe space, caring coaches, and positive socializing are identified as integral and integrated within surf therapy. The surfing component is viewed as a vehicle rather than the focus of the intervention. In the locations selected, surfing was largely a novel sport. The limited surfing that was present was largely due to tourism and was inaccessible for the majority of local communities.

### 2.2. Sample Characteristics

Participants were recruited primarily through local schools or via local community organizations. There was no screening process at this initial pilot stage, and the primary criterion for involvement was referral partners believing individuals would benefit. Each site worked with a different number of participants based on capacity. There was a broad spread of age ranges within the sample (7–22), with an average age of 12.9 years. Attendance was captured using the Teampact app (https://teampact.co/ (accessed on 5 June 2021)), which uses facial recognition and session timers to promote transparency around intervention delivery. Table 1 offers a breakdown of demographics within the surf therapy pilots, including pooled national averages and attendance figures. Site 5 data is not presented due to data collection issues, which are addressed below, but Site 5 had comparable demographics to other sites (*n* = 18, average age 13.7, age range 8–17, 9 male and 9 female).

### 2.3. Mixed Methods Evaluation: Phase 1—Quantitative Analysis

To best pragmatically satisfy the aim of this study, an uncontrolled mixed methods approach was utilized in exploring surf therapy pilot evaluations within Sierra Leone. Phase 1 of the analysis involved statistical analysis of pre/post data to evaluate associated program impacts. To do this, the anonymized pre and post data associated with the pilot projects were made accessible to the research team. The evaluations utilized the Stirling Child Well-Being Scale (SCWS) to measure participant well-being before and after the course. This is a positively worded well-being measure developed by Stirling University in 2010 and has been demonstrated as both psychometrically reliable and valid for children aged 8 and above [13]. The SCWS involves a 12 item positively worded well-being scale, divided into Positive Emotional State and Positive Outlook sub-components. The scale consists of a 5-point Likert format, with totals ranging from 12–60. Higher scores indicate higher well-being. The scale also includes 3 social desirability items to control for response set or socially desirable responses. Such data can be subsequently excluded from analysis.

The anonymous dataset was cleaned to remove any unpaired data, such as pre-tests without corresponding post-tests (6 data items removed). Any scores that fulfilled social desirability criteria on the SCWS were also removed (6 data sets removed). One site (Site 5) did not manage to collect the pre/post data appropriately, and its data was removed. Confusion arose over the anonymous data labelling implemented to protect participant confidentiality. Mislabeling meant it was impossible to track individual attendance and to match pre and post test data. While this was disappointing for this evaluation, it was a valuable learning experience for future data collection.

Given the small sample sizes associated with individual sites, parametric analysis was deemed inappropriate. Wilcoxon signed rank tests were carried out to test for significance of associated impact on well-being. Wilcoxon signed rank tests also allow for exploration of effect size (*r = Z/√n*), to best provide statistical representation of associated pilot outcomes.

### 2.4. Mixed Methods Evaluation: Phase 2—Qualitative Analysis

Phase 2 of the analysis consisted of a synthesis of the published surf therapy evaluations. The primary aim of this synthesis was to supplement the statistical analysis by contextualizing surf therapy in Sierra Leone, while also highlighting the pragmatic challenges that may have had an impact on intervention delivery. Secondary aims included reinforcing associated impact claims through triangulation and highlighting any reported plausible mechanisms for comparison to the wider literature. Original data collection consisted of open-ended qualitative questionnaires provided to all participants and their parents upon culmination of intervention delivery. This questionnaire had a high response rate (Site 1 *n =* 12; Site 2 *n =* 18; Site 3 *n =* 10; Site 4 *n =* 12; Site 5 *n =* 16). An opportunity for open-ended reporting of intervention perceptions was also provided to other community stakeholders, such as local teachers, community leaders, local government officials, police, and healthcare workers. Trends within this original data were identified and reported within the evaluation reports. The evaluations also included subjective project coordinator reports, which captured challenges in delivery and offered interpretations that may not otherwise have been included in evaluations. A combined thematic analysis of the data within the evaluations was conducted to support the study’s aims. These different elements of the evaluations were summarized and tabulated in a descriptive manner in line with the aims of the synthesis.

### 2.5. Mixed Methods Evaluation: Phase 3—Comparative Analysis

Phase 3 of the analysis involved comparing and triangulating findings from Phase 1 and 2 to explore contextualized trends or patterns that would not have been possible if merely considering each phase individually [14]. Further interpretive analysis of findings from both phases, alongside wider literature from within the paradigm, is included within the discussion section to address the study aims [15].

## 3. Results

### 3.1. Phase 1—Statistical Analysis of Well-Being Data

**Figure 1:**
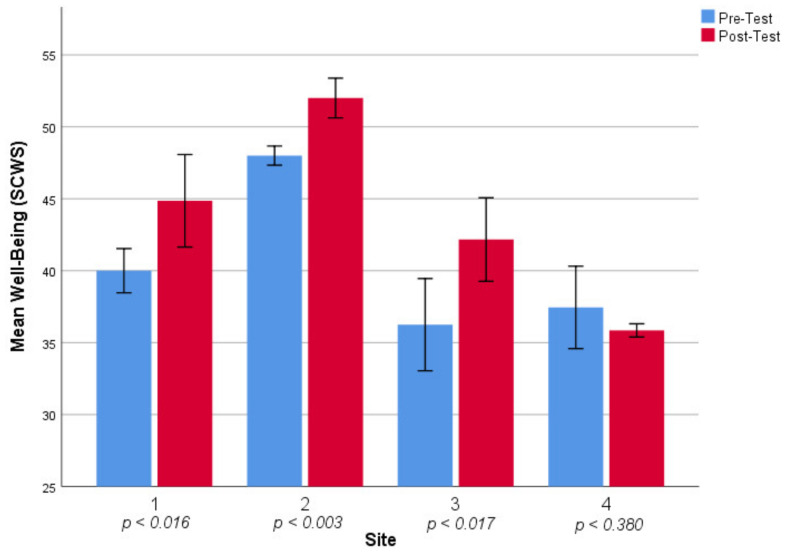
Mean change in participant well-being by site.

Initial statistical analysis consisted of Wilcoxon signed rank tests carried out on the data associated within individual sites. Three of the sites (1,2,3) were associated with statistically significant large positive effects on participant well-being, as summarized in Table 2. Site 4 differed from these other sites and was associated with a statistically non-significant small negative effect on participant well-being (*Z* = −0.88, *p* < 0.380; *r* = −0.22). For ease of comparison, associated impact on participant well-being by site is visualized in Figure 1. Data was not pooled for national analysis, given the significant variation between sites.

### 3.2. Phase 2—Evaluation Synthesis

The evaluation synthesis consisted of summarizing and tabulating key qualitative components of evaluation reports collected from a range of stakeholders, including participants, parents, teachers, healthcare workers, and project coordinators. Summaries were structured in line with synthesis aims: Reported Qualitative Impact, Plausible Mechanisms Reported, and Challenges Reported (shown in Table 3). These identified elements are largely based on individual quotes, and thus definitive conclusions cannot be drawn. Alongside the changes to well-being described by quantitative measures, different types of impact were reported within qualitative data, including improvements to socializing (Sites 1, 3, and, 5) as well as improved mindset and feeling calmer (Sites 1 and 4). Also reported were increased positive behaviors (Site 2) and reductions in negative behaviors (Sites 3 and 5). Another notable trend was the reported impact on academic elements of participants lives, with improvements in attendance (Site 2), effort (Site 4), and performance (Site 3) in school all highlighted by a range of intervention stakeholders, including school teachers.

In relation to plausible mechanisms, a range of program elements emerged across intervention sites, including peer and social support (Sites 1, 2, 3, and, 5), respite (Sites 1 and 5), provision of a safe space (Sites 3 and 4), learned coping skills (Sites 3 and 4), alternatives to negative activities (Site 1), and increased physical activity (Site 5). Given the limited amount of qualitative data and lack of targeted research exploring these plausible mechanisms, findings may not be generalizable, but their presence adds strength to intervention impact claims [16].

One of the most important elements the evaluation synthesis considered was the reported challenges to intervention delivery. There were several themes that emerged across multiple sites, including perceptions of water safety, the need for a feeding component, and the need for transportation. The evaluations listed how these challenges were overcome, largely through sourcing community partnerships, local funding for food/transport provision, and building trust within the community around water safety. One of the sites (Site 4) had a challenging pilot around consistency, including changes to lead coaches and change of location, which may have contributed to or compounded low attendance. This combination of factors may have severely inhibited the associated impact of the program at the pilot stage.

### 3.3. Phase 3—Comparative Analysis

When interpreted in totality, the different phases of analysis offer contextual insight as to the associated impact of surf therapy pilots within Sierra Leone. Three of the sites were associated with significant large positive effects on participant well-being. This associated impact aligned with positive qualitative data that outlined associated positive well-being indicators such as improved socializing, improved behavior, and improved emotional regulation. The evaluation synthesis also highlighted the individual challenges reported at Site 4, which provide context and plausible rational (largely related to low attendance) for why this site was not associated with the same large positive effects as other sites.

## 4. Discussion and Implications

Overall, in this uncontrolled study, three out of four surf therapy sites analyzed were associated with significant large positive effects on participant well-being. These are encouraging initial findings for the piloting of surf therapy in Sierra Leone and support its continued delivery alongside further research. More rigorous research utilizing control groups, blinding, and if possible randomization protocols, are required before any effectiveness claims can be made.

When data were explored by site, the differences of associated impacts observed alongside other intervention data provide valuable insight. Three sites (1,2,3) had comparable significant large positive effects associated with participant wellbeing. From the data collected, the noticeable difference between these sites and Site 4, which had a non-significant associated impact, was attendance figures. Site 4′s attendance was considerably lower than other sites, which suggests a plausible correlation between attendance and associated impact on well-being. Such a correlation may indicate a minimum threshold of exposure to surf therapy that is required before a positive impact can occur. Such a finding aligns with wider research highlighting a dose-response relationship between physical activity and positive mental health outcomes [17]. Other plausible mechanisms highlighted within the evaluation synthesis included the development of social and coping skills, which would take time to transfer and may account for the correlation between attendance and positive impact on well-being. Previous research has highlighted how dosage has an important amplifying effect on activity-based youth development and that further research is required to explore if there are optimal dosages for different kinds of programs [18]. The findings from this initial exploration of surf therapy in Sierra Leone suggest prolonged engagement was key to a positive impact on well-being and should be an important consideration in intervention design. Research to understand the optimal length of program, frequency of delivery, and adherence levels would be beneficial. Further exploration of optimal dosage or possible dose-response relationships within this intervention would be of benefit to both the surf therapy and SfD paradigms, especially given the complete lack of such research within LMICs or post-conflict settings.

The evaluation synthesis also allowed for contextual exploration of other challenges experienced by the interventions at the pilot phase. One site (Site 4) experienced service delivery issues linked to a required change of location and the absence of a lead coach due to a family illness. These challenges, alongside the low attendance, appear to account for the main differences between this site and the other intervention locations. Given the intervention’s theoretical framework includes the premise of a consistently delivered safe space [11], it is perhaps unsurprising that these inconsistencies had the impact they did. Mitigation strategies around similar challenges to consistency should be a priority for all of the intervention sites in the future.

Two further challenges were highlighted by all intervention sites: the need for a feeding component to service delivery and the challenges posed by community beliefs and preconceptions around water safety. All sites reported participants feeling hungry at the end of sessions, which is unsurprising as even small amounts of the kind of paddling associated with surfing have been shown to have significant energy costs [19]. Food security was a reported challenge for some participants and their families. Without including a feeding component, the interventions may have created an additional burden on local communities, which would undermine goodwill and the perceived value of surf therapy. All sites managed to find resources to add feeding components into programming prior to completion of the pilot phase, and this provides an important pragmatic learning for other interventions that include similar levels of energy expenditure.

The other challenge faced by all sites were perceptions/beliefs around water safety. Drowning is a significant health burden across Africa, with children and adolescents being some of the groups at highest risk [20]. The sites overcame these perceptions by building trust and helping to improve understanding around water safety for the whole community rather than just the participants. This included incorporating individuals with aquatic expertise from the local communities into the service delivery, based on recognizing the value of local knowledge and community collaboration [21]. Such individuals came from free-diving or fishing backgrounds and had rescue capability, if not formal rescue qualifications. A perception that further contributed to intervention hesitancy was the notion that surfing could only occur in open or deep water. Practitioners demonstrated to the community that all surf therapy activities happen in standing depth of participants and that building water confidence and swimming skills were included within activity planning. This challenge and the integrated community-based method of overcoming it provide valuable pragmatic learnings for similar intervention designs.

All but one of the sites reported negative changes within well-being to some participants; it is important to understand how this related to the surf therapy interventions, as not doing harm should be a highest priority. Individual negative scores should not be generalized from; the most negative rankings, and therefore scores, were identified within Site 4, which was also observed to have had the lowest attendance figures. This suggests that negative changes to well-being may be related to factors outside of the intervention. As previously discussed, the negative impact of conflict on youth mental health as experienced in Sierra Leone is intergenerational [2] and was further compounded by the 2014–2016 Ebola pandemic [5]. Young people in Sierra Leone still face a wide range of challenges to their mental health in day-to-day life, and these may account for the negative changes reported, especially given their correlation to low attendance.

Plausible mechanisms identified within the evaluation synthesis add strength to claims of the positive impact of surf therapy on participant well-being [16]. Identified mechanisms, including the provision of a safe space, respite, and peer support, align with prior research, which heavily informed the intervention design [11], alongside other research into surf therapy for youth [10,12]. The evidence of participant social and coping skills being recognized by other community stakeholders alludes to the successful implementation of a specific skill transfer model [22]. It should be noted that the intervention exists as a complex intervention, with multiple theoretical mediators [11], and this study does not explore which mediators are most important to achieving outcomes. Future research exploring the mediating effect of different components (e.g., coach characteristics, safe space, social components, coping skills curriculum) within the intervention would be valuable. The triangulation of previously identified mechanisms for youth well-being through surf therapy with intervention stakeholder qualitative data lends further support to the associated positive impact of surf therapy in Sierra Leone.

## 5. Limitations

The most significant limitation of this exploration of surf therapy in Sierra Leone is the lack of any control group to compare intervention groups against. This limits discussion to perceived associated impact and does not allow for interpretation as to the effectiveness of the interventions. While this represents a substantial challenge to the robustness of the findings, the importance of the process that led to them should not be understated. As part of this project the intervention practitioners took ownership over the evaluation process, from data collection through to evaluation write-up. At the evaluation design phase, intervention leadership was offered support from academic partners, who also supported the more in-depth statistical analysis seen within this review. This process supported the recognized interests and wishes of SfD practitioners to better understand and be able to conduct their own evaluations [21]. The aim of this process was also recognizing and addressing concerns around neocolonialist approaches to research within the SfD field by prioritizing the development of individual and organizational evaluation capacity as highly as any other evaluation outcome [21].

Future work can build upon this even further with continued capacity building and locally led research. This work carried out by the surf therapy practitioners deserves to be recognized and shared, especially the honest reporting of challenges faced and, at times, the non-significant impact. This process also provides the platform for future, locally owned, more rigorous research that may have otherwise not been possible. Intervention coordinators are exploring the potential of a randomized controlled trials (RCTs) of surf therapy in the future, which would allow for an exploration of intervention effectiveness that was impossible in this current work. While the challenges of conducting an RCT within a surf therapy intervention are considerable, especially around the difficulties of blinding different arms, a locally led waitlist protocol would be a desirable piece of research for the surf therapy, SfD, and blue space paradigms [23].

The SCWS is a validated and recognized tool for measuring child and youth well-being [13]. Despite its successful use in multiple studies, its genesis within a Western context (Scotland) should be recognized. The challenges of the use of and the predominance of Western mental health tools in different contexts have been acknowledged by researchers [24]. The process of developing and validating locally informed well-being and mental health measurements has been previously reported [25], and a similar process to generate a Sierra Leone–specific measurement would be of significant benefit to future research in the region. Exploring the physiological measurements associated with well-being, such as heart rate variability or cortisol, may also offer different ways of exploring or further triangulating the impact of surf therapy in Sierra Leone.

## 6. Conclusions

This uncontrolled mixed methods study highlights promising initial findings related to the positive associated impact of surf therapy for youth well-being in Sierra Leone across multiple sites and supports continued delivery. The findings highlight the importance of prolonged participant engagement with surf therapy alongside the value of working together with local communities in overcoming challenges in intervention delivery. Lessons learned in the piloting of surf therapy in Sierra Leone offer pragmatic insight into intervention delivery while providing the foundations and initial steps for locally owned more rigorous research in the future.

## Figures and Tables

**Table 1:** Sample demographics.

Site Number	Organization	*n*	Average Age	Age Range	Average Attendance	Male	Female
na	Pooled National Data	58	12.9	7–22	57.20%	35	23
1	Moseray Fadika Trust	14	13.3	9–20	89.30%	2	10
2	Young Leaders Sierra Leone	12	15.1	11–18	59.50%	12	0
3	United Sierra Leone	12	12.3	7–16	58.30%	11	3
4	Pipul Pikin Foundation	20	11.7	8–22	37.20%	10	10

**Table 2:** Breakdown of changes to well-being in Sierra Leone surf therapy.

Site	*Z*	Asymp. Sig *(p)*	Effect Size (*r*)
1	−2.415	0.016	0.65
2	−2.944	0.003	0.84
3	−2.395	0.017	0.69
4	−0.877	0.380	−0.22

**Table 3:** Summary of qualitative reporting within surf therapy evaluations.

Intervention Site	Reported Qualitative Impact	Plausible Mechanisms Reported	Challenges Reported
1	Feeling calmer, Improved socializing	New positive relationships, Respite from stress, Alternative to negative activities	Perceptions around water safety, Need for food within program, Lack of disability access, Transport to program
2	Improved academic attendance, Behavioral improvements	Peer support from other surfers, Positive social relationships	Perceptions around water safety, Need for food within program, Lack of female participation, Transport to program
3	Improved socializing, Reduction in bad behaviors, Academic performance	Peer support from coaches, Safe space, Coping skills	Perceptions around water safety, Need for food within program, Accessibility to younger (<8) participants
4	Improved mindset, Improved academic effort	Coping skills, Safe Space	Consistency of delivery, Low attendance, Change of location, Change of lead coaches, Perceptions around water safety, Need for food within program
5	Improved socializing, Reduction in bad behaviors	Respite, Peer support from coaches, Increased physical activity	Evaluation confusion, Perceptions around program aims

## Data Availability

The data presented in this study are available on request from the corresponding author: james.marshall@napier.ac.uk.

## Appendix A. Links to Surf Therapy Pilot Evaluations

Moseray Fadika Trust Foundation: https://www.waves-for-change.org/wp-content/uploads/2020/08/MFT-Evaluation-Report-Final.pdf (accessed on 5 June 2021).

Young Leaders Sierra Leone: https://www.waves-for-change.org/wp-content/uploads/2020/07/Young-Leaders-Sierra-Leone-Surf-Therapy-Report.pdf (accessed on 5 June 2021).

United Sierra Leone: https://www.waves-for-change.org/wp-content/uploads/2020/07/United-Sierra-Leone-Sierra-Leone-Surf-Therapy-Report.pdf (accessed on 5 June 2021).

Pipul Pikin Foundation: https://www.waves-for-change.org/wp-content/uploads/2020/07/Pipul-Pikin-Sierra-Leone-Surf-Therapy-Report.pdf (accessed on 5 June 2021).

Job Opportunities for Youth Sierra Leone (JOY): https://www.waves-for-change.org/wp-content/uploads/2020/08/JOY-SIERRA-LEONE-SURF-THERAPY-REPORT.pdf (accessed on 5 June 2021).
